# Quantifying COVID-19 Content in the Online Health Opinion War Using Machine Learning

**DOI:** 10.1109/ACCESS.2020.2993967

**Published:** 2020-05-11

**Authors:** Richard F. Sear, Nicolás Velásquez, Rhys Leahy, Nicholas Johnson Restrepo, Sara El Oud, Nicholas Gabriel, Yonatan Lupu, Neil F. Johnson

**Affiliations:** 1 Department of Computer ScienceGeorge Washington University 8367 WashingtonDC20052USA; 2 Institute for Data, Democracy, and Politics, George Washington University 8367 WashingtonDC20052USA; 3 Elliott School of International AffairsGeorge Washington University 8367 WashingtonDC20052USA; 4 ClustrX LLCWashingtonDC20007USA; 5 Department of PhysicsGeorge Washington University 8367 WashingtonDC20052USA; 6 Department of Political ScienceGeorge Washington University 8367 WashingtonDC20052USA

**Keywords:** COVID-19, machine learning, topic modeling, mechanistic model, social computing

## Abstract

A huge amount of potentially dangerous COVID-19 misinformation is appearing online. Here we use machine learning to quantify COVID-19 content among online opponents of establishment health guidance, in particular vaccinations (“anti-vax”). We find that the anti-vax community is developing a less focused debate around COVID-19 than its counterpart, the pro-vaccination (“pro-vax”) community. However, the anti-vax community exhibits a broader range of “flavors” of COVID-19 topics, and hence can appeal to a broader cross-section of individuals seeking COVID-19 guidance online, e.g. individuals wary of a mandatory fast-tracked COVID-19 vaccine or those seeking alternative remedies. Hence the anti-vax community looks better positioned to attract fresh support going forward than the pro-vax community. This is concerning since a widespread lack of adoption of a COVID-19 vaccine will mean the world falls short of providing herd immunity, leaving countries open to future COVID-19 resurgences. We provide a mechanistic model that interprets these results and could help in assessing the likely efficacy of intervention strategies. Our approach is scalable and hence tackles the urgent problem facing social media platforms of having to analyze huge volumes of online health misinformation and disinformation.

## I. Introduction

Scientific experts agree that defeating COVID-19 will depend on developing a vaccine. However, this assumes that a sufficiently large proportion of people would receive a vaccine so that herd immunity is achieved. Because vaccines tend to be less effective in older people, this will require younger generations to have very high COVID-19 vaccination rates in order to guarantee herd immunity [1]. Yet there is already significant opposition to existing vaccinations, e.g. against measles, with some parents already refusing to vaccinate their children. Such vaccine opposition increased the number of cases in the 2019 measles outbreak in the U.S. and beyond [2]. Any future COVID-19 vaccine will likely face similar opposition [3], [4]. Mandatory COVID-19 vaccinations for schoolchildren could trigger a global public health conflict. A better understanding of such opposition ahead of a COVID-19 vaccine is therefore critical for scientists, public health practitioners, and governments.

Online social media platforms, and in particular the built-in communities that platforms like Facebook (FB) feature, have become popular fora for vaccine opponents (anti-vax) to congregate and share health (mis)information. Such misinformation can endanger public health and individual safety [1], [4]. Likewise, vaccine supporters (pro-vax) also congregate in such online communities to discuss and advocate for professional public health guidance. Well before COVID-19, there was already an intense online conflict featuring anti-vax communities and pro-vax communities. Within anti-vax communities, the narratives typically draw on and generate misinformation about establishment medical guidance and distrust of the government, pharmaceutical industry, and new technologies such as 5G communications [1], [4], [5]. Adding fuel to this fire, the January 2020 birth of the COVID-19 “infodemic” has led to a plethora of misinformation in social media surrounding COVID-19 that directly threatens lives [6]. For example, harmful “cures” are being proposed such as drinking fish tank additives, bleach, or cow urine, along with coordinated threats against public health officials like Dr. Anthony Fauci, director of the U.S. National Institute of Allergic and Infectious Diseases [7]. Moreover, false rumors have been circulating that individuals with dark skin are immune to COVID-19. These may have contributed to more relaxed social distancing among some minorities and hence their over-representation as victims. In Chicago and Louisiana as of early April 2020, ~70% of the fatalities were African Americans even though this demographic only makes up ~30% of the population [8], [9]. In addition, the world has witnessed an alarming rise in COVID-19 weaponization against the Asian community [10]–[12]. It is also clear that such misinformation is not a fringe phenomenon, and can instead be very widely held as true within the general population. Indeed, a recent Pew study [13] found that ~30% of Americans believe the COVID-19 virus was likely created in a laboratory, despite statements from infectious disease experts to the contrary.

Unfortunately, the sheer volume of new online content and the speed with which it spreads, means that social media companies are struggling to contain such health misinformation [14], [15]. Making matters worse, people around the world are spending more time on social media due to social distancing imposed during the COVID-19 pandemic. This increases the likelihood that they become exposed to such misinformation, and as a result they may put themselves and their contacts at risk with dangerous COVID-19 remedies, cures and falsehoods.

The present study is motivated by both these needs: (1) the need for a deeper understanding of this intersection between online vaccination opposition and the online conversation surrounding COVID-19; and (2) the need for an automated approach since the sheer volume of new online material every day makes manual analysis a non-viable option going forward. We pursue an automated, machine learning approach that avoids the scalability limitations of manual content analysis. While the present paper is just the first step in a challenging longer-term goal, the automated approach that we present allows the following questions to be addressed: How did COVID-19 change the online conversation within anti-vaccination and pro-vaccination communities over the two month period in early 2020 when the disease became a global threat; and what do the topical changes that we observe in the anti-vax and pro-vax online communities’ narratives, imply about their relative abilities to attract new supporters going forward?

Unlike many existing works, this study does not use Twitter data [16], [17] since it is known that Twitter is more of a broadcast medium for individual shout-outs, whereas discussions and narratives tend to be nurtured in in-built online community spaces that are a specific feature of platforms like Facebook (e.g., fan page) [18]. Twitter does not have such in-built community spaces. In the present methodology, generalized from [19] and [20], data is collected from these online communities, specifically Facebook Pages that support either anti-vaccination or pro-vaccination views. This information is publicly available and does not require any individual details, thereby avoiding any privacy concerns – just as understanding the content of a conversation among a crowd of people in an open, real-world public space does not require knowledge of any personal details about the individuals within that crowd. Details of our approach are given in Sec. II and the Appendix. A third difference between this study and previous ones is that the machine learning findings here are interpreted in terms of a mechanistic model (Sec. IV) that captures the general trend for the coherence in the online conversations over time. Though much work still needs to be done, this study therefore provides a first step toward a fully automated but interpretable understanding of the growing public health debate concerning vaccines and COVID-19.

## II. Data and Machine Learning Analysis

The terms ‘Facebook Page’ and ‘cluster’ are used interchangeably here since each Facebook Page is a cluster of people. Facebook Pages, also known as fan pages or public pages, are accounts that represent organizations, causes, communities, or public figures. According to Facebook’s policies, “Content posted to a Page is public and can be viewed by everyone who can see the Page” [see 21, Sec. 5]. A Facebook Page is different from a Facebook personal account. Personal accounts represent private individuals, and their posts and interactions are considered more private and targeted to their immediate contacts. This paper does not analyze data from personal accounts. Our methodology follows [19] and [20] by analyzing the public content of Facebook Pages for both anti-vaccination (“anti-vax”) and pro-vaccination (“pro-vax”) communities. The publicly available content of these online communities is obtained using a snowball approach, starting with a seed of manually identified pages discussing either vaccines, public policies about vaccination, or the pro-vs-anti vaccination debate. Then their connections to other fan pages are indexed. At each step, new clusters are evaluated through a combination of human coding and computer-assisted filters. To classify a cluster as being (1) anti-vax or pro-vax and (2) including COVID-19 content or not, we reviewed its posts and the Page’s “about” section. Pro-vax and anti-vax classifications required that either (a) at least 2 of the most recent 25 posts dealt with the pro-vax or anti-vax debate, or (b) the page’s title or “about” section described it as pro-vax or anti-vax. At least two researchers classified each cluster independently. If they disagreed on their suggested classification, a third researcher reviewed the posts and then all three reviewers discussed these cases. Agreement was reached in each case. This also enabled us to distinguish between content that is intended to be serious versus merely satirical. The self-weeding tendency within Facebook Pages tends to reduce material from bots and fake profiles. We kept the present study focused on English, though this can be easily generalized using our same procedure. Beyond that, our study was global and not limited to a particular region.

The content of these clusters was then bundled together separately for the anti-vax community and the pro-vax community, and the two resulting sets of content were analyzed using machine learning. Specifically, we used an unsupervised machine learning technique called Latent Dirichlet Allocation (LDA) [22] to analyze the emergence and evolution of topics around COVID-19. The LDA method models documents as distributions of topics and topics as distributions of words. During its training process, these distributions are adjusted to fit the dataset. The LDA method is described correctly in Wikipedia as [23] “[quote].. a generative statistical model that allows sets of observations to be explained by unobserved groups that explain why some parts of the data are similar. For example, if observations are words collected into documents, it posits that each document is a mixture of a small number of topics and that each word’s presence is attributable to one of the document’s topics. LDA is an example of a topic model and belongs to the machine learning toolbox and in wider sense to the artificial intelligence toolbox.”

The coherence score provides a quantitative method for measuring the alignment of the words within an identified topic (see [22]). It is generated from a separate algorithm which is run over a trained LDA model. The overall coherence score of a single model is the arithmetic mean of its per-topic coherences. There are many different coherence metrics to evaluate per-topic coherence. We use $C_{\mathrm {V}}$ which is based on a sliding window, one-set segmentation of the top words and an indirect confirmation measure that uses normalized point-wise mutual information and the cosine similarity. It comprises collections of probability measures on how often top words in topics co-occur with each other in examples of the topics. We refer to [22] for a full explanation and discussion of $C_{\mathrm {V}}$.

Machine learning automation can, in principle, help address the significant issues facing social media platforms by mechanically picking out material that requires attention from the huge haystack of online content. While this could help to better curtail online misinformation, one might rightly ask about its accuracy and reliability as compared to human analysts. This has been recently addressed in [24]. We use the same coherence metric ($C_{\mathrm {V}}$) as these authors. They addressed the problem that topic models had previously given no guarantee on the interpretability of their output. Specifically, they produced several benchmark datasets with human judgements of the interpretability of topics and they found results that outperformed existing measures with respect to correlation to human ratings. They achieved this by evaluating 237,912 coherence measures on 6 different benchmarks for topic coherence, making this the biggest study of topic coherences at that time. Separately, we have done our own comparison for the general area of online hate and have found comparable consistency.

In summary, our machine learning approach identifies topics in the online narratives with high coherence, meaning the word groupings identified are strongly related according to the coherence scoring approach discussed earlier. Our human inspection of the word distribution making up each grouping showed that they do indeed correspond to reasonably distinct conversation topics. Details and examples are given in the Appendix.

## III. Results

The main focus here is in the endogenous development of COVID-19 conversation at the beginning of the global pandemic and prior to the first officially reported U.S. COVID-19 death on February 29, 2020 [25]. Hence we collected Facebook public post data for the period 1/17/2020-2/28/2020 inclusive. To assess the change over time, this period was divided into time intervals. Since having more time intervals would mean smaller amounts of data within each and hence more fluctuations, and since we are just interested in the change over time, two intervals were chosen of equal duration, $T_{1}$ and $T_{2}$. The first time-interval 1/17/2020-2/7/2020 ($T_{1}$) contains 774 total pro-vax posts and replies, and 3630 total anti-vax posts and replies. The second time-interval 2/7/2020-2/28/2020 ($T_{2}$) contains 673 total pro-vax posts and replies, and 3200 total anti-vax posts and replies. Hence our two equal time windows contains similar amounts of data. We checked that our results are relatively robust to other choices of time interval. Interestingly, $T_{1}$ roughly corresponds to the time when COVID-19 was largely seen as a problem in Asia, while $T_{2}$ roughly corresponds to the time during which it became a serious problem in Europe. For further reassurance that our data was representative of the COVID-19 conversation during these intervals, we also checked that the data split is similar to that for mentions of COVID-19 in article counts from worldwide anglophone newspapers and worldwide Google trends.

**FIGURE 1.**
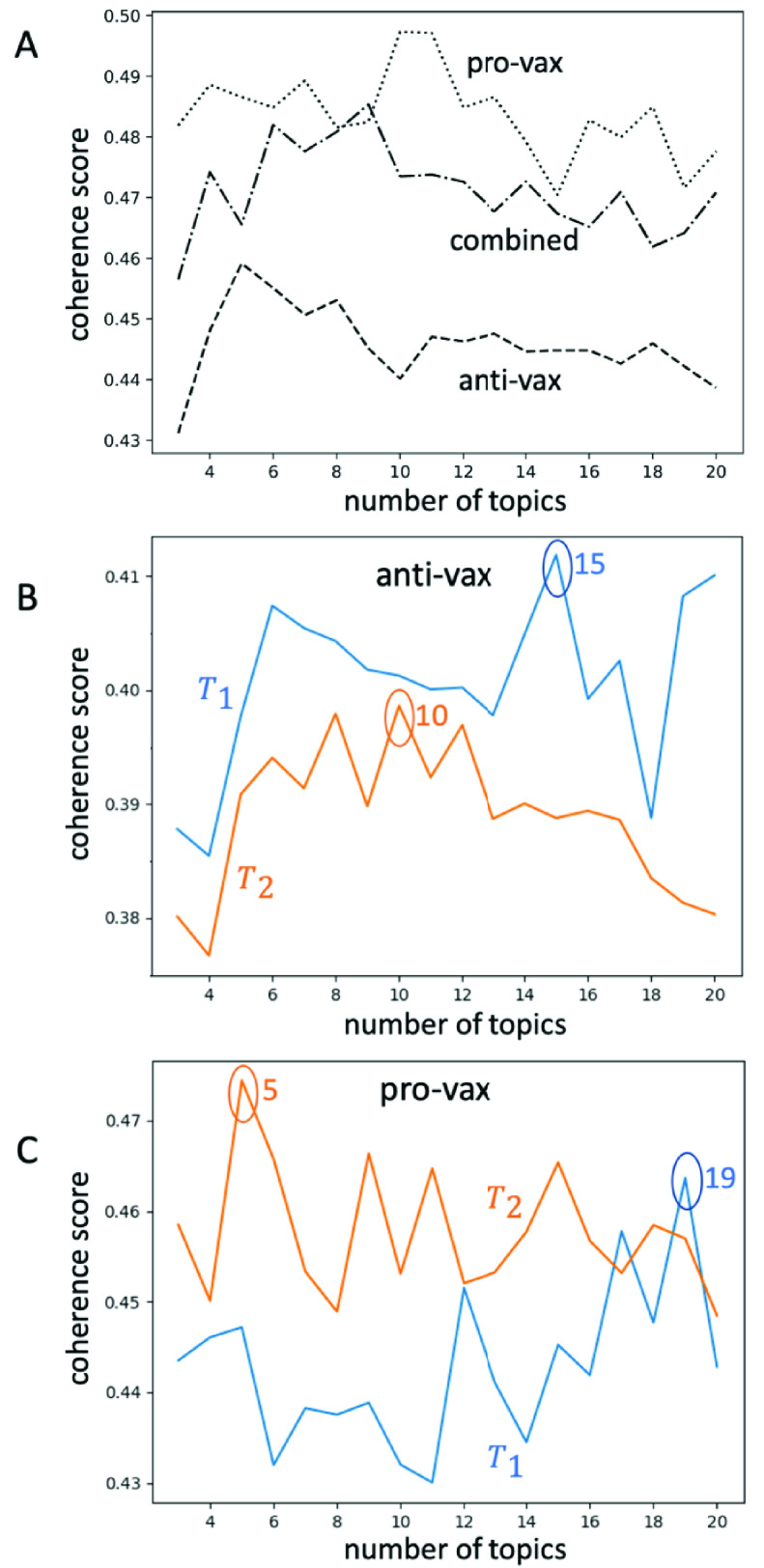
Coherence scores $C_{\mathrm {V}}$ for (A) anti-vax (dashed line), pro-vax content (dotted line), and anti-vax combined with pro-vax (dashed-dotted line), calculated over the entire time period of study ($T_{1}+T_{2}$). (B) Anti-vax content for the separate time periods $T_{1}$ (blue line) and $T_{2}$ (orange line). The number of topics for which the coherence score $C_{\mathrm {V}}$ is a maximum is indicated, i.e. the optimal number of topics. The optimal number of topics for anti-vax moves from 15 to 10 from $T_{1}$ to $T_{2}$. (C) Pro-vax content for the separate time periods $T_{1}$ (blue line) and $T_{2}$ (orange line). The optimal number of topics for pro-vax moves from 19 to 5 from $T_{1}$ to $T_{2}$.

The LDA models were trained over posts in the following distinct groups: anti-vaccination posts in $T_{1}$, anti-vaccination posts in $T_{2}$, pro-vaccination posts in $T_{1}$, and pro-vaccination posts in $T_{2}$. For each of these sets, 10 separate LDA models were trained with the *number of topics* parameter ranging from 3-20, for a total of 180 models in each of the four groups. Fuller details are given in the Appendix. The $C_{\mathrm {V}}$ coherence algorithm was then run over each of these models and the coherence scores were averaged for each number of topics. These averaged scores are plotted in Figures 1B and 1C. Figure 1A shows the result of the same procedure applied to all posts in our dataset, and to all anti-vaccination posts, and to all pro-vaccination posts.

The coherence score $C_{\mathrm {V}}$ for the entire period of study (i.e. $T_{1}+T_{2}$) in Fig. 1 A, is consistently larger across the number of topics for pro-vax than for anti-vax, suggesting that the pro-vax community overall has a more focused discussion around COVID-19 than the anti-vax. This is consistent with the pro-vax community featuring a more monolithic discussion around public health – namely, it is focused on advising people to follow professional medical guidance.

The bad news for the pro-vax community from this higher overall coherence, is that it is less well positioned to engage with the wide variety of more blurry, and often more extreme, COVID-19 narratives that are now circulating online. This represents a significant potential disadvantage for the pro-vax community in that it may therefore be less able to attract the attention of the many different types of users who are now entering this online space in search of a particular nuanced ‘flavor’ of COVID-19 narrative that appeals to them. These users could consequently be pulled toward the anti-vax cause.

Figures 1B and 1C indicate the change over time by comparing the curves of the coherence score across number of topics, for time periods $T_{1}$ and then $T_{2}$. The curve moves up from $T_{1}$ to $T_{2}$ for the pro-vax community (Fig. 1C) and the optimal number of topics shows a dramatic decrease from 19 to 5. This is consistent with the notion that the pro-vax community is working toward a common COVID-19 interpretation and narrative with fewer ‘flavors’ of discussion and interpretation than the anti-vax community. Again, while this may sound like a strength, it suggests that the pro-vax community overall is actually becoming *less* appealing over time to the many different types of new users who are in search of their own COVID-19 narrative ‘flavor’. By contrast, the curves for the anti-vax community from $T_{1}$ to $T_{2}$ (Fig. 1B) show a far smaller reduction in the optimal number of topics (15 to 10) and the curves move down, in the opposite direction to the pro-vax. Hence the anti-vax compensates a small increase in focus (reduction in the optimal number of topics) with an overall reduction in coherence, i.e. these 10 topics for $T_{2}$ are effectively more blurry than the original 15 for $T_{1}$, and hence the overall anti-vax community is becoming more accommodating to the diverse population of new additions coming into the online health space over time.

**FIGURE 2.**
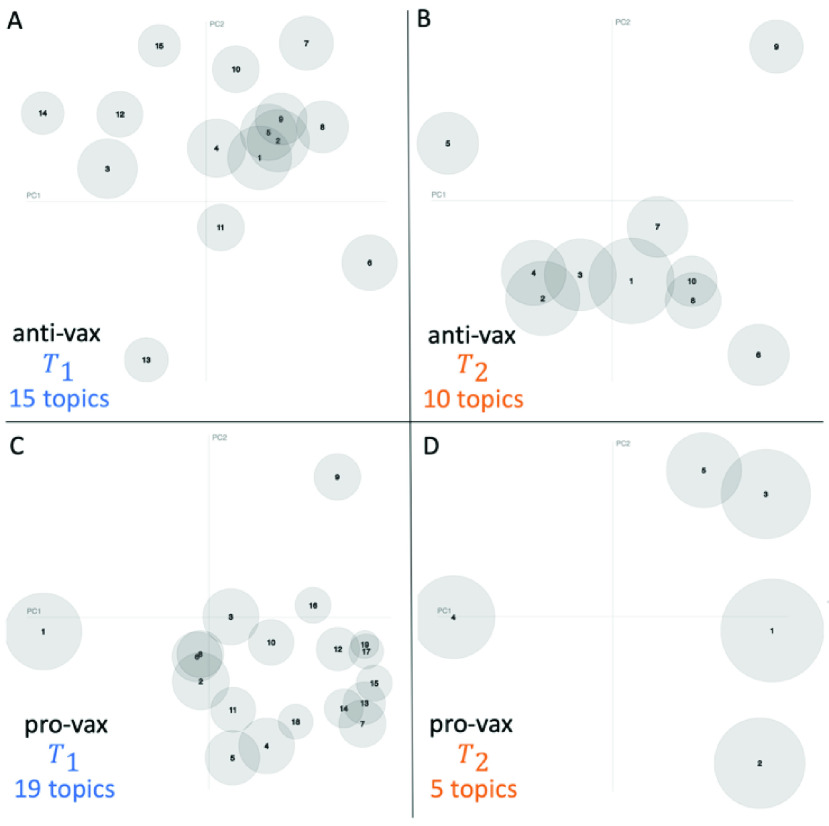
Visualization of the informational structure of the individual topics, and how they relate to each other. This plot is obtained using pyLDAvis. The circles in each plot are the topics from Fig. 1 for which the average coherence score is highest, i.e. the optimal number of topics. Their size indicates the marginal topic distribution as discussed in detail in [26], while the two axes are principal components in the distribution analysis.

Figure 2 shows a visualization with more detail about the information structure of the individual topics, and how far these topics are from one another in terms of informational distance. The plot is obtained using the pyLDAvis package [26] which provides a global view of the topics and how they differ from each other, while at the same time allowing for a deeper inspection of the terms most highly associated with each individual topic. This provides a novel method for implying the relevance of a term to a topic. The study in [26] showed that ranking terms purely by their probability under a topic, by contrast, is suboptimal for topic interpretation. We refer to [26] for full details of LDAvis.

The change in the pro-vax community from time period $T_{1}$ (Fig. 2C) to $T_{2}$ (Fig. 2D) is such that the optimal number of topics decreases (i.e., the number of circles decreases from 19 to 5 following Fig. 1C) and the topics evolve to become located mostly in the same portion of the space (i.e., toward the right-hand side of Fig. 2D). Following Fig. 1B, the change in the anti-vax community from time period $T_{1}$ (Fig. 2A) to $T_{2}$ (Fig. 2B) is such that the optimal number of topics starts off slightly smaller than the pro-vax, but although it also decreases over time (i.e., the number of circles decreases) there are more topics (i.e., more circles in Fig. 2B) than for the pro-vax in time period $T_{2}$ (Fig. 2D). Also, the topics seem more spread out across the space in Fig. 2B as compared to Fig. 2D. These observations are consistent with our earlier interpretations that the pro-vax community is more focused (equivalently, narrower) than the anti-vax community in terms of COVID-19 narratives, and that the pro-vax community is evolving toward a common COVID-19 interpretation and narrative with a lower diversity on offer than the anti-vax community.

## IV. Toward a Mechanistic Model Interpretation

**FIGURE 3.**
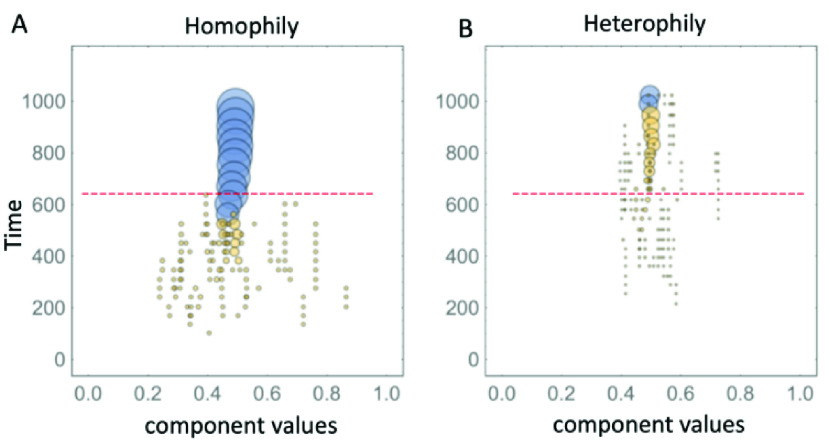
Output from our mechanistic model in which clusters form if the component ${x}$-values are sufficiently similar (i.e., homophily in panel A) or different (i.e., heterophily in panel B).

We created a mechanistic model that further supports these empirical findings and provides a microscopic interpretation of the machine learning output. Specifically, we generated a computer simulation of an ecology of online components of the overall community content, each of which is characterized by a vector $\boldsymbol {x}=(x_{1}$, $x_{2}$,…) in which each component $x_{i}$ signifies the strength of a given factor surrounding the online health debate, e.g. government control. The exact nature of these components does not need to be specified, i.e. whether they are words or short phrases. It just matters that there is a diverse ecology of such building blocks. This mechanistic model setup, while seemingly very simplistic, does indeed reflect the empirical observations and literature surrounding the themes of online discussions of vaccination opposition, as listed and studied in detail by Kata in [1]. We then carry out a simulation whereby these components are selected randomly to build up content. Components cluster together (or their clusters cluster together, if they are already in a cluster) if their ${x}$ values are sufficiently similar (i.e. homophily in Fig. 3A) or different (i.e. heterophily in Fig. 3B). To illustrate the output of our model, Fig. 3 shows a one-dimensional version. We checked that a two-dimensional version gives similar results, though it is visually more complicated because of having the time component along the third dimension. Most importantly, it produces plots that are visually similar to those in Fig. 2. As can be seen from Fig. 3, the case of homophily (which is akin to building a more monolithic topic discussion with few flavors, like the pro-vax community) has a convergence that is quicker, as observed in Figs. 1 and 2 for the pro-vax community. By contrast, the case of heterophily (which is akin to building diverse topic discussions with many flavors, like the anti-vax community) is slower to gel, which is consistent with the anti-vax community in Figs. 1 and 2. The red dotted horizontal line in Figs. 3A and 3B gives an indication of the stage in the simulation that is broadly consistent with Figs. 2D and 2B for the pro-vax and anti-vax communities respectively.

The delay in the gelation time observed in Fig. 3B for heterophily (anti-vax) as compared to homophily (pro-vax) in Fig. 3A, can be derived analytically using mathematical analysis from statistical physics (see [27] for full details). In particular, we have been able to show that the time at which gelation emerges depends inversely on the average probability that two randomly picked components join the same cluster, which is smaller for heterophily than homophily and hence the gelation time is later for heterophily than homophily – exactly as observed in Fig. 3. Similarly, it can be shown mathematically that the gelation sizes (akin to the sizes of the circles in Fig. 2) will be smaller for heterophily than homophily, as also observed in Fig 3.

Again, instead of this being good news for the pro-vax community, the simulation of this mechanistic model shows that the case of homophily (pro-vax) is less able to absorb an influx in new users with a range of ${x}$ values, as compared to the case of heterophily (anti-vax). This is consistent with the idea stated earlier, that the anti-vax community appears more engaging to new users (e.g. parents with children of school-age who are wary of school vaccine requirements, or who fear government control) and hence the anti-vax will be more able to gain new supporters in the long run than the pro-vax.

## V. Limitations of the Study

There are of course many limitations of this study. There are other social media platforms, apart from Facebook, that should be explored – but Facebook is the largest. Similar behaviors should arise in any platform where communities can form. It will also be interesting, for example, to compare our findings to other studies focused on Twitter, where messaging is more in the form of short, individual statements [17]. There is also the question of influence of external agents or entities [16]. However, these social media communities tend to police themselves for bot-like or troll behavior. Further analysis is required of the details of the content. This will require going beyond just text and perhaps beyond LDA, since memes and images are also shared. Also, the generative model output needs to be compared in detail to the time-evolution of topics. Further research is also required to formulate the results across all platforms into detailed, actionable consequences for policy makers. These limitations will be addressed in future work.

## VI. Conclusion

These findings suggest that the online anti-vax community is developing a more diverse and hence more broadly accommodating discussion around COVID-19 than the pro-vax community. As a result, the pro-vax community runs the risk of making itself less engaging to the heterogeneous ecology of potential new users who join the online COVID-19 discussion, and who may arrive online with a broad set of concerns, questions, and possibly preconceived notions, misinformation and even falsehoods.

The analysis in this paper also provides a first step toward eventually either replacing, or at least supplementing, the non-scalable efforts of human moderators tasked with identifying online misinformation. In addition, the mechanistic model (Fig. 3) could be used for what-if scenario testing of how quickly coherence develops and what the impact would be of breaking up the coherence around certain topics, e.g. by counter-messaging against individuals ingesting bleach or the even newer ‘COVID Organics’ that are circulating as a cure in Madagscar, Africa and beyond. This can be achieved by using the empirical analysis in Fig. 2 – repeated over multiple consecutive time intervals – to identify the growth of topics around new words which may be gaining popularity as a home cure (e.g. “bleach”). Then Facebook, for example, could post ads that specifically target these specific new words and topics, rather than blanket vanilla messaging promoting establishment medical science narratives.

Overall, this approach shows that a machine-learning algorithm, the LDA algorithm, identifies plausible topics within collections of posts from online communities surrounding the vaccine and COVID-19 debate. In addition to being able to handle large quantities of data, its results emerge quickly using statistical grouping techniques, instead of having to rely on potentially biased, slow and costly human labeling.

## Appendix

As mentioned in the main text, the methodology starts with a seed of manually identified Facebook Pages discussing either vaccines, public policies about vaccination, or the pro-vs-anti vaccination debate. Then their connections to other fan pages are indexed. At each step, new findings are vetted through a combination of human coding and computer assisted filters. This snowball process is continued, noting that new links can often lead back to members already in the list and hence some form of closure can in principle be achieved. This process leads to a set containing many hundreds of pages for both the anti-vax and pro-vax communities. Before training the LDA models, several steps are employed to clean the content of these pages in a similar way to other LDA analyses in the literature:

Step 1: Mentions of URL shorteners are removed, such as “bit.ly” since these are fragments output by Facebook’s CrowdTangle API.
Step 2: Many of the posts link to external websites. The fact that these specific websites were mentioned could itself be an interesting component of the COVID-19 conversation. Hence instead of removing them completely, the pieces “.gov”, “.com”, and “.org” were replaced with “__gov”, “__com”, and “__org”, respectively. This operation effectively concatenates domains into a form that will not be filtered out by the later preprocessing steps.
Step 3: The posts are then run through Gensim’s simple_preprocess function, which tokenizes the post on spaces and removes tokens that are only 1 or 2 characters long. This step also removes numeric and punctuation characters.
Step 4: Tokens that are in Gensim’s list of stopwords, are removed. For example, “the” is not a good indication of a topic.
Step 5: Tokens are lemmatized using the WordNetLemmatizer from the Natural Language Toolkit NLTK, which converts all words to singular form and/or present tense.
Step 6: Tokens are stemmed using the SnowballStemmer from NLTK, which removes affixes on words.
Step 7: Any remaining fragments of URLs (other than domain) that are left over after stemming, such as “http” and “www”, are removed.

Steps 5 and 6 help ensure that words are compared fairly during the training process, and that if a particular word is a strong indicator of a topic, its signal is not lost just because it is used in many different forms. These steps rely on words existing in NLTK’s pretrained vocabulary. Any word not in this vocabulary is left unchanged. After this preprocessing, we then train the LDA models on the cleaned data. Specifically, 10 separate LDA models were trained with the “number of topics” parameter ranging from 3-20, for a total of 180 models in each of the two time intervals $T_{1}$ and $T_{2}$. The $C_{V}$ coherence algorithm was then run over each of these models and the coherence scores were then averaged for each number of topics. To produce the results, multiple trials were run for each number of topics to ensure that the coherence for a particular number of topics is representative of what LDA models tend to find (and by extension a better fit for the data) and is not the result of unaccounted noise swaying the model to overfit in one way or another. These trials are independent because the random number generator for each LDA model was initialized with a different seed, ensuring that statistical inferences were not be repeated. The GitHub link is: https://github.com/searri/social-clustering-research/wiki/Coronavirus-Vax.

The following illustrates the topic output, focusing on anti-vax in time interval $T_{2}$ in Fig. 2 B. In this, 9 of the 10 topics had the word ‘coronavirus’ among the 5 highest weighted words in the topic; 4 were focused around ‘coronavirus’ and ‘vaccine’ co-occuring together. Others had ‘vitamin’, ‘fear’ and ‘ddt’ in relation to alternative treatments, and ‘weapon’ related to conspiracy theories of COVID-19’s origin. Within one of the topics, which is focused around alternative health explanations and cures with words like ‘vitamin’ etc., illustrative posts include the following from Feb 8, 2020 in one of the ‘Coalition for Vaccine Choice’ pages, with spelling mistakes left as is: “The story of this FAKE “epidemic” with the “corona virus” from China is a cover-up story for the grim reality of the health problems due to 5G technology exposure coroborated with a lot of other factors: vaccination, poor alimentation in vitamins, bad water, air pollution, lack of sleep, etc…. scientists have shown that low level microwave EMF exposure can result in VGCC activation and elevated intracellular calcium”. Meanwhile, for a topic focused on conspiracy theories with words such as ‘weapon’ and ‘fear’, an example phrase from a posting is “..keeping the world under the thumb of tyrants! You are soldiers, and that means that you are expendable by your trained nature. You are being micro-managed by people that give not one caring thought of you, five thousand miles away, that know little of the true nature of the battle”. This illustrates the type of detailed analyses that we carried out to check our automated approach, and which underlie our claim that the groupings do correspond to reasonably distinct conversation topics.
